# Supplementary material for: Coral endosymbiont growth is enhanced by metabolic interactions with bacteria
Source: Nat Commun. 2023 Oct 27;14:6864. doi: 10.1038/s41467-023-42663-y (PMC10611727; doi:10.1038/s41467-023-42663-y)
Supplement: Supplementary file 1 — Supplementary Information [file 41467_2023_42663_MOESM1_ESM.pdf]

Supplemental Information

for

Coral endosymbiont growth is enhanced by metabolic interactions with bacteria

Jennifer L. Matthews[1*], Abeeha Khalil[1], Nachshon Siboni[1], Jeremy Bougoure[2], Paul Guagliardo[2], Unnikrishnan Kuzhiumparambil[1], Matthew DeMaere[1], Nine M. Le Reun[1], Justin R. Seymour[1], David J. Suggett[1] and Jean-Baptiste Raina[1]

[1]Climate Change Cluster, University of Technology Sydney, Ultimo NSW 2007, Australia

[2]Centre for Microscopy, Characterisation and Analysis, University of Western Australia, Perth WA 6009 Australia.

*Corresponding author: Jennifer Matthews, Jennifer.Matthews@uts.edu.au, Climate Change Cluster (C3), University of Technology Sydney, PO Box 123 Broadway NSW 2007 AUSTRALIA. Phone: +61 02 9514-4087, Fax: +61 02 9514-4079

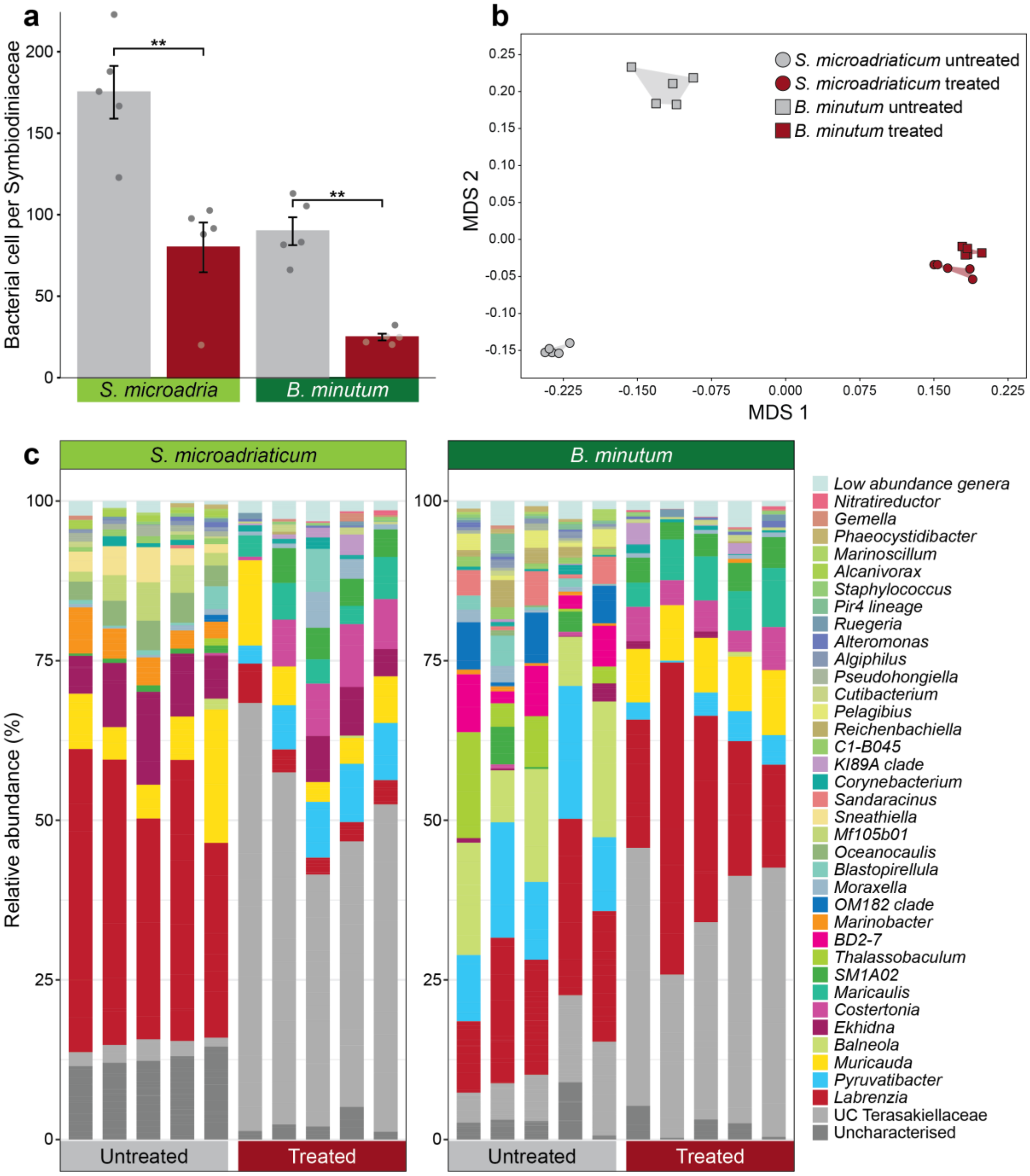

Figure S1. Microbial composition and comparison between control and Ab+Tx-treated *Symbiodinium microadriaticum and Breviolum minutum* cultures. (A) Comparison of the total bacterial abundance per Symbiodiniaceae cell of untreated (grey) and Ab+Tx treated (maroon) cultures (Two way T-test, ** = $p < 0.01$). (B) nMDS ordination of *Symbiodinium microadriaticum* (circles) and *Breviolum minutum* (squares) coloured according to treatment, based on a Bray–Curtis similarity matrix of rarefied ASV abundance data (Supplementary Data 3). (C) Bacterial community composition (relative abundance %) for untreated and Ab+Tx treated Symbiodiniaceae strains. Sequences were processed using QIIME framework v2.2018.6[50] Taxonomy was assigned using *classify-sklearn*[53] against the SILVA v138 database. Source data are provided as a Source Data file.

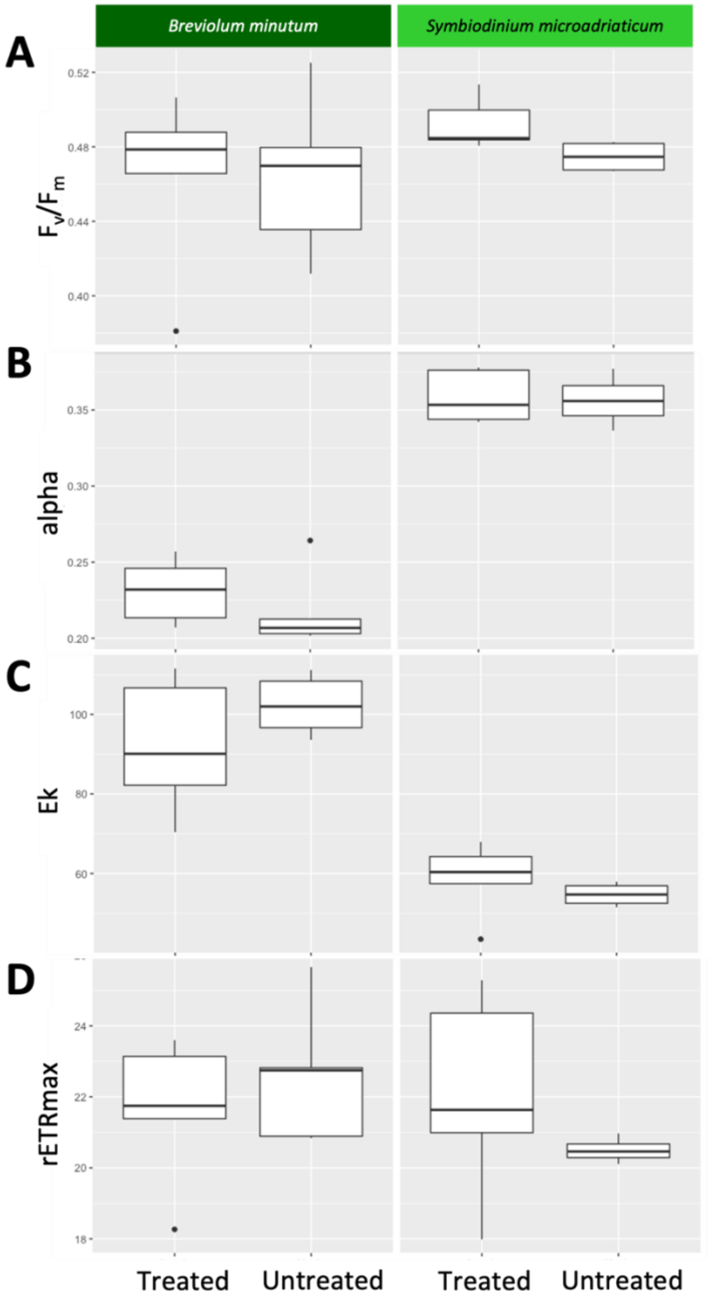

Figure S2. Effects of antibiotic treatment on Symbiodiniaceae photophysiology. Boxplots compare the A) quantum yield (F_v/F_m); B) maximum light use efficiency (alpha); C) optimal light condition (Ek); and D) the maximum relative rate of electron transport ($rETR_{max}$) between AbTx-treated and untreated cultures of *Breviolum minutum* (left) and *Symbiodinium microadriaticum* (right) (*n* = 5 per treatment per species). Source data are provided as a Source Data file.

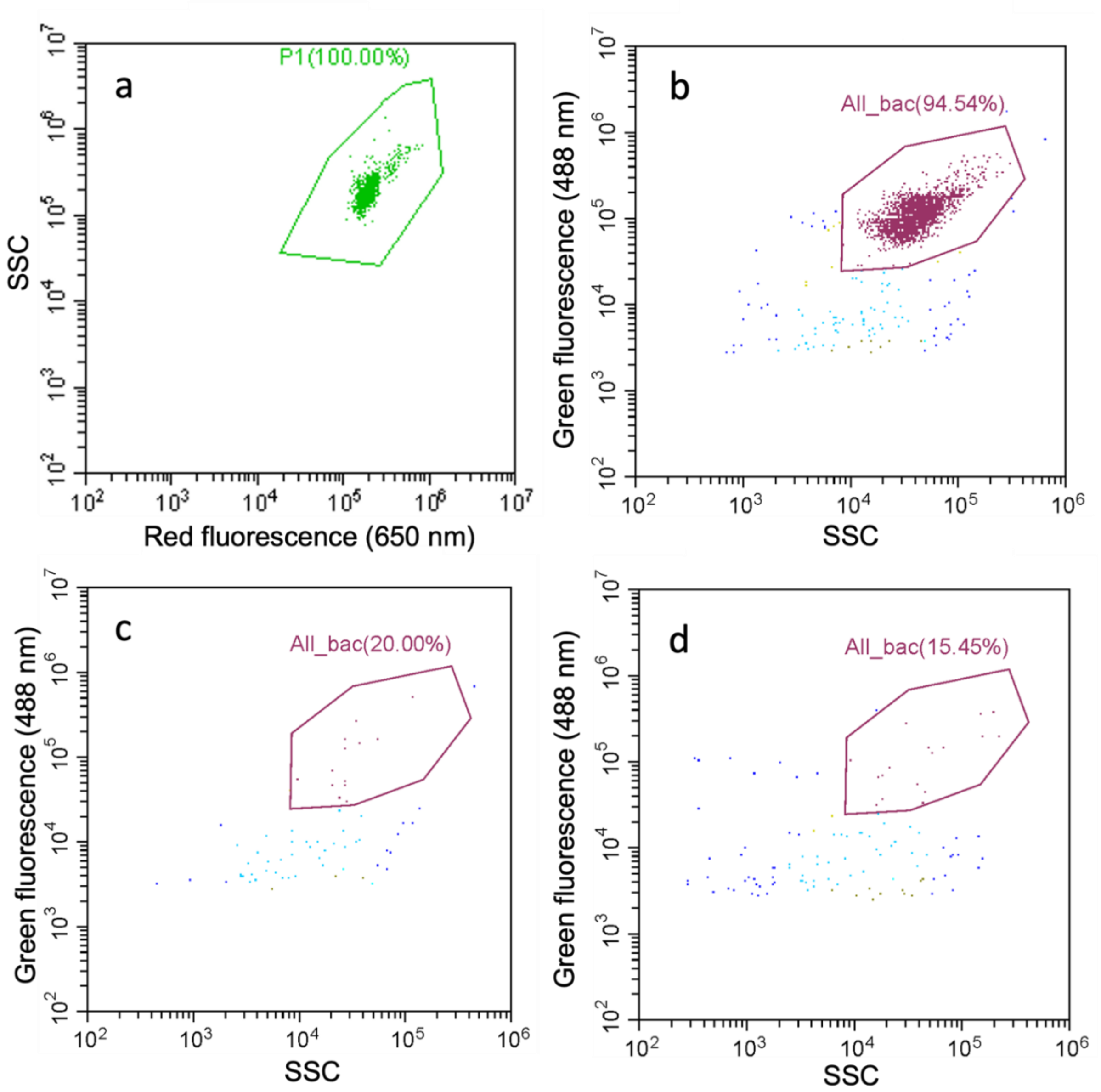

Figure S3: Gating strategy used to collect flow cytometry data. (a) Bacteria population characterized according to side scatter (SSC) and SYBR Green fluorescence. **(b)** Symbiodiniaceae sp. population characterized according to side scatter (SSC) and red fluorescence (chlorophyll). **(c)** Example bacteria blank (media only) flow cytometry results used for blank corrections. **(d)** Example of EBR treated culture flow cytometry results.

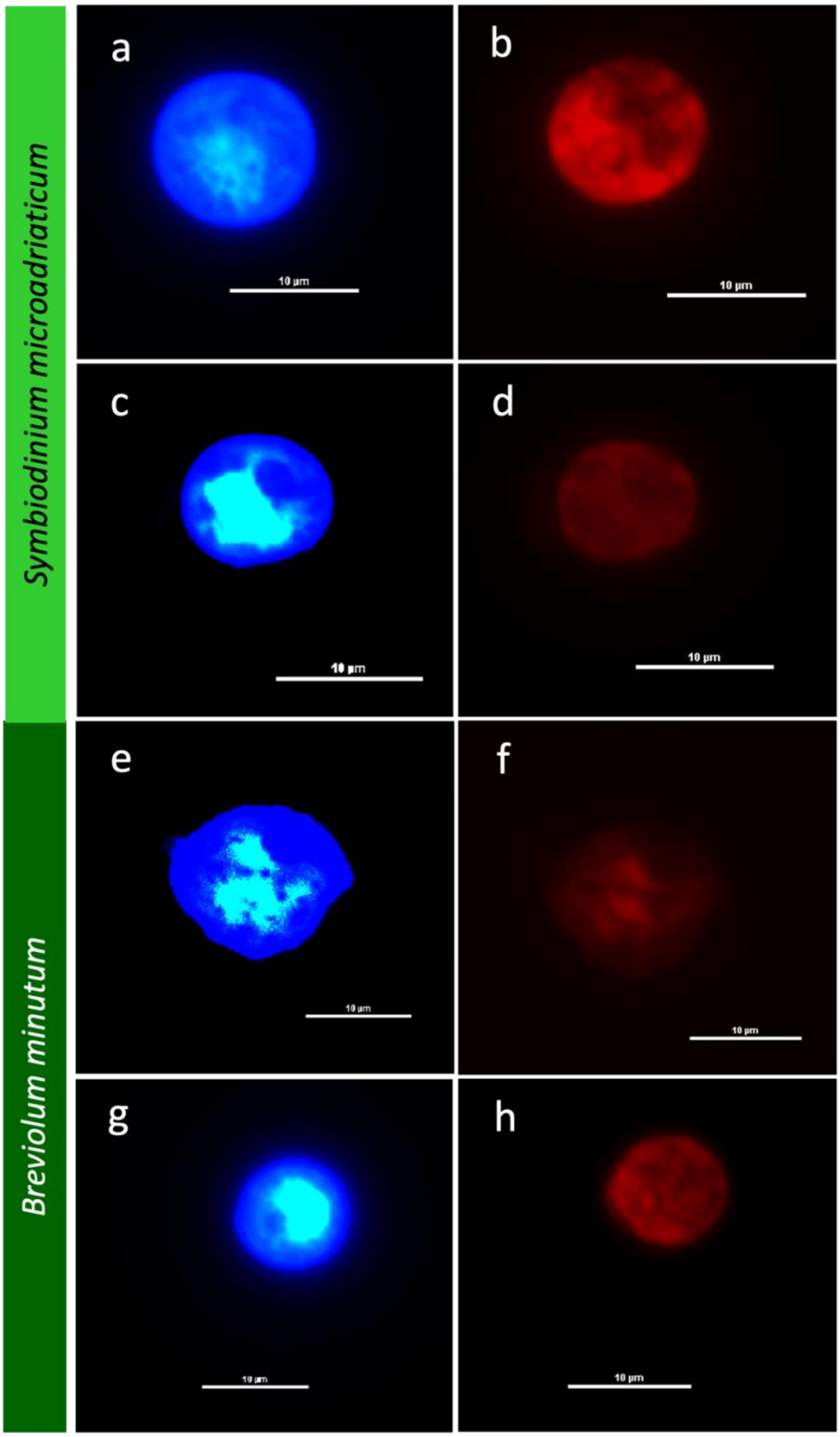

Figure S4. Microscopy verification of the EBR culture treatment. DNA DAPI staining (blue) and Symbiodiniaceae chlorophyll autofluorescence (red) of the EBR cultures of *S. microadriaticum* (a-d) and *B. minutum* (e-h). *n* = 20 cells per treatment per species were viewed.

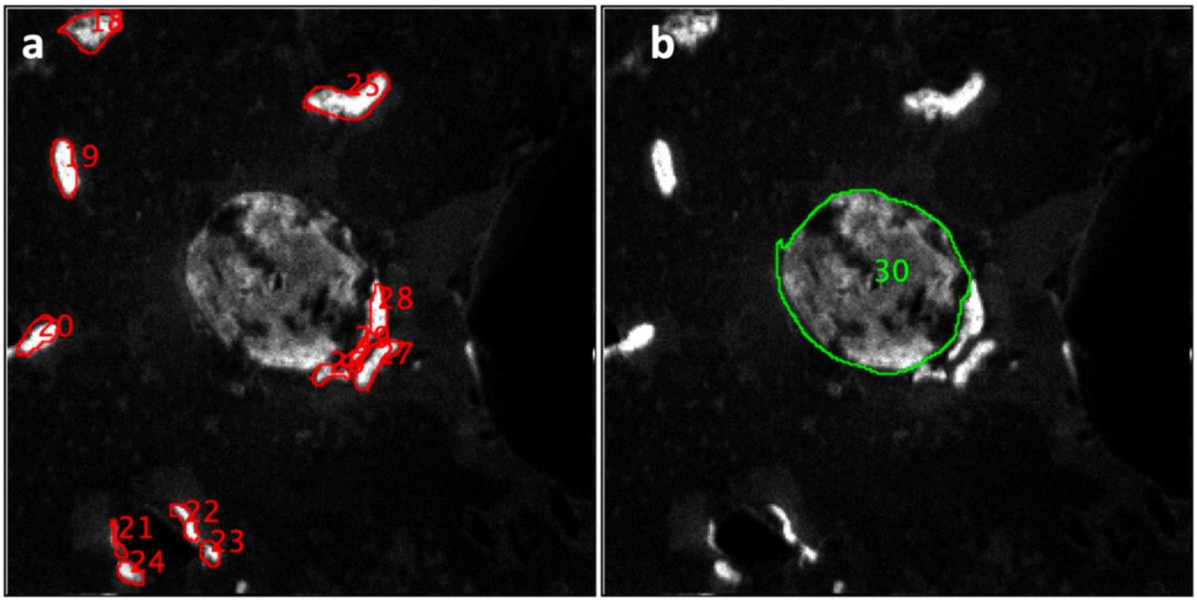

Figure S5: Region of interest strategy used to select cell regions for isotope abundance analysis. Regions of interest (ROIs) were selected based on the silhouette of the **(a)** Bacteria and **(b)** Symbiodiniaceae sp. cells from the $^{12}C^{14}N^-$, using the ImageJ plugin OpenMIMS.
