# Supplementary material for: Coral endosymbiont growth is enhanced by metabolic interactions with bacteria
Source: Nat Commun. 2023 Oct 27;14:6864. doi: 10.1038/s41467-023-42663-y (PMC10611727; doi:10.1038/s41467-023-42663-y)
Supplement: Supplementary file 4 — Reporting Summary [file 41467_2023_42663_MOESM4_ESM.pdf]

nature portfolio

Corresponding author(s): Jennifer Matthews

Last updated by author(s): Oct 4, 2023

Reporting Summary

Nature Portfolio wishes to improve the reproducibility of the work that we publish. This form provides structure for consistency and transparency in reporting. For further information on Nature Portfolio policies, see our Editorial Policies and the Editorial Policy Checklist.

Statistics

For all statistical analyses, confirm that the following items are present in the figure legend, table legend, main text, or Methods section.

n/a	Confirmed	
☐	☒	The exact sample size (*n*) for each experimental group/condition, given as a discrete number and unit of measurement
☐	☒	A statement on whether measurements were taken from distinct samples or whether the same sample was measured repeatedly
☐	☒	The statistical test(s) used AND whether they are one- or two-sided<br>*Only common tests should be described solely by name; describe more complex techniques in the Methods section.*
☐	☒	A description of all covariates tested
☐	☒	A description of any assumptions or corrections, such as tests of normality and adjustment for multiple comparisons
☐	☒	A full description of the statistical parameters including central tendency (e.g. means) or other basic estimates (e.g. regression coefficient) AND variation (e.g. standard deviation) or associated estimates of uncertainty (e.g. confidence intervals)
☐	☒	For null hypothesis testing, the test statistic (e.g. *F*, *t*, *r*) with confidence intervals, effect sizes, degrees of freedom and *P* value noted<br>*Give P values as exact values whenever suitable.*
☒	☐	For Bayesian analysis, information on the choice of priors and Markov chain Monte Carlo settings
☒	☐	For hierarchical and complex designs, identification of the appropriate level for tests and full reporting of outcomes
☐	☒	Estimates of effect sizes (e.g. Cohen's *d*, Pearson's *r*), indicating how they were calculated

Our web collection on statistics for biologists contains articles on many of the points above.

Software and code

Policy information about availability of computer code

Data collection	CytExpert Version 2.4, Agilent MassHunter Qualitative and Quantitative Analysis version B.08.00, LabSolutions Insight, Illumina MiSeq platform (2 × 300 bp). All LIFT-FRRf (Soliense Inc., USA) programming and model fitting was conducted using a custom-designed application software package (https://soliense.com/LIFT_Method.php, accessed 21 December 2021).
Data analysis	Fiji Version 1.53c with Open-MIMS plug-in, statistics were carried out in R v4.1.1 or PAST v4.03, and all analysis scripts will be available on Zeondo (10.5281/zenodo.8385031.)

For manuscripts utilizing custom algorithms or software that are central to the research but not yet described in published literature, software must be made available to editors and reviewers. We strongly encourage code deposition in a community repository (e.g. GitHub). See the Nature Portfolio guidelines for submitting code & software for further information.

Data

Policy information about availability of data

All manuscripts must include a data availability statement. This statement should provide the following information, where applicable:

- Accession codes, unique identifiers, or web links for publicly available datasets
- A description of any restrictions on data availability
- For clinical datasets or third party data, please ensure that the statement adheres to our policy

Bacterial sequences for the isolates of Labrenzia, Muricauda and Marinobacter used have been uploaded to NCBI BioProject PRJNA922609. A reporting summary for

this article is available as a Supplementary Information file. All data supporting our findings have been provided in the Supplementary Data files. Source data are provided with this paper.

Human research participants

Policy information about studies involving human research participants and Sex and Gender in Research.

Reporting on sex and gender	This is not human research
Population characteristics	This is not human research
Recruitment	This is not human research
Ethics oversight	This is not human research

Note that full information on the approval of the study protocol must also be provided in the manuscript.

Field-specific reporting

Please select the one below that is the best fit for your research. If you are not sure, read the appropriate sections before making your selection.

☐ Life sciences ☐ Behavioural & social sciences ☒ Ecological, evolutionary & environmental sciences

For a reference copy of the document with all sections, see nature.com/documents/nr-reporting-summary-flat.pdf

Ecological, evolutionary & environmental sciences study design

All studies must disclose on these points even when the disclosure is negative.

Study description	This study aimed to characterise if bacteria provide metabolite resources to the coral endosymbiont Symbiodiniaceae when free-living. We carried out bacteria composition analysis, metabolomics, NanoSIMS, and co-growth experiments, on two Symbiodiniaceae species when untreated, with no extracellular bacteria, and in co-culture with bacteria species isolated from the Symbiodiniaceae (Labrenzia alexandrii, Marinobacter sp. and Muricauda aquimarina). Each experiment was replicated. The experiments were either one- or two-factor designs.
Research sample	The research samples consisted of subcultures of two species of Symbiodiniaceae (Symbiodinium microadriaticum RT61, and Breviolum minutum RT2) that were able to be rendered bacteria free. Populations originated from long term laboratory stocks. These species were co-cultured with and without bacteria isolated from the Symbiodiniaceae cultures, Labrenzia alexandrii, Marinobacter sp. or Muricauda aquimarina.
Sampling strategy	Pilot studies were carried out before conducting the experiment described in this manuscript. We selected our sample size and Symbiodiniaceae species that provided minimum variability, maximised feasibility of handling within a short time frame required for metabolomics analysis based on these pilot studies, and previous studies that examined bacteria-phytoplankton interactions (Amin, S. et al. Interaction and signalling between a cosmopolitan phytoplankton and associated bacteria. Nature 522, 98; 2015). No statistical method was used to predetermine sample size.
Data collection	Bacteria amplicons were sequenced using the Illumina MiSeq platform (2 × 300 bp) at the Australian Genome Research Facility (Melbourne, Australia). Gas Chromatography mass spectra were collected by JL Matthews electronically using a 72030 Shimadzu gas chromatograph and a TQ8050 quadrupole mass spectrometer (Shimadzu, Japan). Cell counts data were recorded by JL Matthews using CytExpert Version 2.4. Stable isotope enrichment data were collected on a NanoSIMS 50 (Cameca, Gennevilliers, France) at the Centre for Microscopy, Characterisation and Analysis (CMCA) at The University of Western Australia by J Bougoure and P Guagliardo. Liquid Chromatography mass spectra were collected electronically by U Kuzhiumparambil using a Shimadzu LCMS-8060 (Shimadzu, Kyoto, Japan) instrument containing a dual ion source (DUIS) interfaced to the Shimadzu Nexera X2 liquid chromatography system.
Timing and spatial scale	Symbiodiniaceae cultures were subculture for one month prior to treatment (08/02/22 - 08/03/22). Symbiodiniaceae photophysiology, bacteria composition and metabolomic samples were collected six days after antibiotic treatment (14/03/22). Symbiodiniaceae and bacteria cell counts were conducted every 1-3 days (from 14/03/22- 31/03/22). IAA concentration was collected from pure bacteria isolates 11 h after inoculation into media broth (24/04/22), when cells were late-exponential growth (determined from pilot studies). For stable isotope tracing (04/05/22), samples were collected after 36 hours, based on pilot studies. For co-culture experiments, growth data were collected every third day for 14 days (the length of the growth cycle for these Symbiodiniaceae cultures, 1/04/22-14/4/22). Symbiodiniaceae and bacteria samples were all derived from original stock populations at the University of Technology Sydney.
Data exclusions	No data was excluded from the study and analyses.
Reproducibility	All experiments described in the manuscript were fully replicated, with four or more biological replicates.
Randomization	Samples were allocated into groups based on the co-culture treatment identity (i.e., either (1) untreated; (2) extracellular bacteria

	removed; (3) co-cultured with Labrenzia alexandrii, Marinobacter sp. or Muricauda aquimarina)
Blinding	Blinding was not pertinent to our study because it did not include any animals and/or human research participants. In addition, blinding was not possible since many analyses were also carried out by the persons in charge of sampling.

Did the study involve field work? ☐ Yes ☒ No

Reporting for specific materials, systems and methods

We require information from authors about some types of materials, experimental systems and methods used in many studies. Here, indicate whether each material, system or method listed is relevant to your study. If you are not sure if a list item applies to your research, read the appropriate section before selecting a response.

Materials & experimental systems

n/a	Involved in the study
☒	☐ Antibodies
☒	☐ Eukaryotic cell lines
☒	☐ Palaeontology and archaeology
☒	☐ Animals and other organisms
☒	☐ Clinical data
☒	☐ Dual use research of concern

Methods

n/a	Involved in the study
☒	☐ ChIP-seq
☐	☒ Flow cytometry
☒	☐ MRI-based neuroimaging

Flow Cytometry

Plots

Confirm that:

☒ The axis labels state the marker and fluorochrome used (e.g. CD4-FITC).

☒ The axis scales are clearly visible. Include numbers along axes only for bottom left plot of group (a 'group' is an analysis of identical markers).

☒ All plots are contour plots with outliers or pseudocolor plots.

☒ A numerical value for number of cells or percentage (with statistics) is provided.

Methodology

Sample preparation	An aliquot of 100 µL was collected from each 1 mL sample of Symbiodiniaceae culture, diluted 1:10 and directly used for flow cytometry analysis (CytoFLEX S, Beckman Coulter, CA, United States) to assess Symbiodiniaceae cell concentration by relative cell chlorophyll fluorescence.<br>Total prokaryotic abundances were quantified by staining the cells with SYBR Green (1:10,000 final dilution). For each sample of each culture, 3 × 200 µL aliquots were taken at the time of sampling and fixed in glutaraldehyde (Sigma-Aldrich; 2% final concentration), and incubated for 15 mins in the dark before analysis. The samples were analysed at a flow rate of 25 µL min–1, with bacterial cells discriminated according to forward scatter (FSC), side scatter (SSC), and green fluorescence (SYBR Green).
Instrument	CytoFLEX S (Beckman Coulter, USA) using filtered MilliQ water as sheath fluid.
Software	CytExpert Version 2.4
Cell population abundance	The target bacteria and Symbiodiniaceae cell populations were the only ones present in the analysis plots.
Gating strategy	Gating around the populations were determined by pilot studies.

☒ Tick this box to confirm that a figure exemplifying the gating strategy is provided in the Supplementary Information.
